# Quantification of supercolonial traits in the yellow crazy ant, *Anoplolepis gracilipes*

**DOI:** 10.1093/jis/14.1.25

**Published:** 2014-01-01

**Authors:** Benjamin D. Hoffmann, Henry Hagedorn

**Affiliations:** CSIRO, Ecosystem Sciences, Tropical Ecosystems Research Centre, PMB 44, Winnellie, NT 0822, Australia

**Keywords:** colony, invasion, nest fidelity, polygyny, supercolony, resource flow

## Abstract

Supercoloniality is a social structure displayed by many invasive ant species, but there has been surprisingly little research quantifying the extent to which individual species display traits underlying such social organisation. This study quantifies three traits for the yellow crazy ant, *Anoplolepis gracilipes* Smith (Hymenoptera: Formicidae): little or no aggression between workers from different nests; the exchange of workers among nests; and resource exchange among nests, as well as supercolony structure arising from patterns of distribution and density of detections. Supercolonies displayed a structural continuum from being small ( < 10 ha) and “aggregated” with great continuity among detections through to being large (>10,000 ha) and “diffuse” with little continuity among detections. Smaller supercolonies had greater ant densities than larger supercolonies. In laboratory trials, no aggression was observed between workers from different nests sourced from different supercolonies, and paired nests merged within 24 hours. Workers lacked nest fidelity by rapidly populating artificial nests containing alien queens. The daily worker turnover rate per nest was estimated to be below 20%. Resources were readily moved among nests, with a resource being detected up to 13 m away from a source within 24 hours, and as far as 32 m after four days. The rate and distance of resource movement increased with increasing worker and nest density. This research has demonstrated that *A. gracilipes* displays supercoloniality equivalent to that of the well-studied Argentine ant *Linepithema humile* . Quantification of these traits is required for other supercolonial species to improve our understanding of this social strategy, especially for invasive ants to aid in understanding factors that promote invasion success and to improve management.

## Introduction

The study of supercoloniality (large-scale polygyny-polydomy) in ants is of great research importance not just for our understanding of eusociality, but because the extreme forms of supercoloniality are dis-proportionally displayed by invasive ants ( Chapman and Bourke 2001 ; Holway et al. 2002 ; Krushelnycky et al. 2010 ). Originally, there were clear definitions of supercoloniality ( Wilson 1971 ; Bourke and Franks 1995 ), but it is now clear that this social organization is not so easily categorised, and single concepts do not exist even for a species ( Le Breton et al. 2004 ; Pedersen et al. 2006 ; Buczkowski 2010 ). Instead, there is a continuum of social types ( LeBrun 2010 ; Steiner et al. 2010 ). Consequently, there is great non-uniformity in the definitions of key words used when discussing supercoloniality. In the case studies presented here, a supercolony is explicitly referred to as an individual population that is geographically, and thus reproductively, isolated from others ( *sensu*Hoffmann et al. 1999 ; Krushelnycky et al. 2005 ; Hoffmann and Saul 2010 ; Thomas et al. 2010 ). Thus, a geographically discrete population is a supercolony. Multiple discrete populations within the study region that are genetically indistinct and do not display aggression when experimentally brought into contact ( Gruber et al. 2012 ) are specifically not referred to as being one supercolony ( *sensu*Buczkowski et al. 2004 ; Jaquiéry et al. 2005 ; Suhr et al. 2009 ).

Most research into supercoloniality has focused on causes and consequences ( Fornier et al. 2009 ; Vogel et al. 2009 ). In contrast, surprisingly little work has quantified the extent to which species display traits that underlie supercoloniality, such as levels of polygyny, low intraspecific aggression, low nest fidelity, dispersal of resources among local nests, and budding. As a result, our understanding of the expression of supercoloniality, and thus the distinction between the multiple forms of supercoloniality ( Heller 2004 ; Pedersen et al. 2006 ; Suarez et al. 2008 ; Helanterä et al. 2009 ), is based upon a handful of studies focused on only a few select species. For example, of the supercolonial species that are invasive, only for the Argentine ant, *Linepithema humile* , has it been demonstrated that workers move among nests ( Markin 1968 ). It can only be assumed for the other invasive species that there is an exchange of workers among nests due to worker trails between nests, or the dispersal of a traceable resource throughout a populated area, which could also be attributed to trophallaxis ( Mortreuil and Brader 1962 ).

Only by quantifying the expression of traits underlying supercoloniality for numerous species will we be able to determine the relative importance of each trait to the level of supercoloniality, and potentially to invasiveness ( Holway and Suarez 1999 ). For example, the exchange of workers, resources, or genes among local nests are thought to be basic internal dynamics within supercolonies, but it appears that nest inter-connectedness does not necessarily translate to the flow of these factors. It was recently demonstrated for a single population of *L. humile* , the most extreme supercolonial species known, that all nests did not interact as a single cooperative unit, but rather a population cluster existed that did not share workers or resources with other nests ( Heller et al. 2008 ). Some non-invasive species of *Formica* displaying supercoloniality have distinct genetic variation among nests, suggesting high nest fidelity ( Chapuisat and Keller 1999 ; Holzer et al. 2006 ). Likewise, the polygyne form of the invasive red imported fire ant, *Solenopsis invicta* , also has low nest relatedness among neighboring colonies ( Goodisman et al. 2007 ). Combined with knowledge that foraging of polygyne *S. invicta* is constrained by neighboring nests ( Weeks et al. 2004a ) and that few resources are shared among nests and only within a very restricted distance ( Drees et al. 1992 ; Weeks et al. 2004b ), workers of this species clearly do not free-flow among nests, and thus the polygyne form of *S. invicta* can only be considered to be partially supercolonial.

The use of thresholds of such traits could also be useful to make a definitive distinction between species that are merely polydomous and polygynous versus those that form supercolonies and ultimately can become unicolonial ( Pedersen et al. 2006 ; Suarez et al. 2008 ; Helanterä et al. 2009 ). For example, in urban areas the odorous house ant, *Tapinoma sessile* , forms polygynous-polydomous colonies displaying great polygyny, but the number of nests per colony as well as the area inhabited by colonies is relatively small compared to many other polydomous species ( Buczkowski 2010 ). Likewise, polygynous-polydomous colonies of *L. humile* in its native range can be as small as tens of metres ( Pedersen et al. 2006 ). So, given the subjective criteria of a supercolony being a population so large that direct interactions between workers from separate nests becomes impossible ( Helanterä et al. 2009 ), can the small colonies of both of these species described above even be considered to be supercolonial?

Like many invasive ant species, the yellow crazy ant, *Anoplolepis gracilipes* Smith (Hymenoptera: Formicidae), can form large-scale (>100 ha) supercolonies ( Haines and Haines 1978a ; Abbott 2005) . This ant has invaded numerous locations of intact and relatively homogenous bushland in remote northern Australia ( Hoffmann and Saul 2010 ), with all spatially discrete supercolonies comprising a single haplotype ( Gruber et al. 2012 ). This scenario allows as far as practicable the study of replicate supercolonies, not confounded by genetic differences, habitat, and human-mediated disturbance. The present study quantifies this ant’s expression of three functional traits that allow multiple nests to function as a single unit, and hence underpin supercoloniality. Three questions are addressed: (1) Do workers from different nests and supercolonies display aggression towards each other? (2) Do workers move among nests, and if so, to what extent? (3) Are resources actively dispersed within a supercolony, and if so, how far and at what rate?

**Figure 1.**
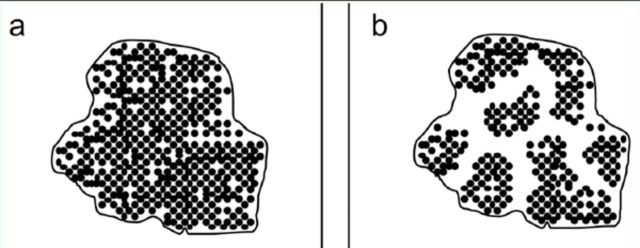
Spatial representations of an *Anoplolepis gracilipes* supercolony (a) with all nests comprising a single cluster and (b) comprising multiple clusters of nests. High quality figures are available online.

Internal supercolony structure arising from patterns of distribution and density is also investigated. Investigations into structural components of supercolonies are few and are mostly restricted to only a segment of the overall supercolony, such as plots of nests ( Heller 2004 ; Heller et al. 2008 ) or segments of boundaries ( Ingram 2002 ; Menke and Holway 2006 ). Only for non-invasive species has the structure of an entire supercolony been documented ( McIver et al. 1997 ; Storer et al. 2008 ). Specifically, the present study tests whether supercolonies are comprised of a single contiguous unit of nests ( Figure 1a ) or multiple clusters of nests ( Figure 1b ) in the absence of perceived abiotic constraints.

## Materials and Methods

### Study area

All research was conducted near Nhulunbuy in northeast Arnhem Land (12°11’S, 136°46’E) in Australia’s Northern Territory. The regional climate is tropical monsoonal with high temperatures (17–33°C) throughout the year and an annual rainfall of approximately 1200 mm falling predominantly during the summer wet season from December to July. The landscape is dominated by xeric savanna woodland with little topography, providing uniform climatic conditions throughout the region.

### Supercolony structure

Supercolony structure, as displayed by *A. gracilipes* patterns of occurrence, was quantified for 14 geographically discrete supercolonies. These supercolonies inhabited an identical vegetation type (savanna woodland) in locations with little (if any) varying topography and with no perceived abiotic or biotic constraints. Mapping was conducted between 2005 and 2009. Visual assessments of the presence/absence of *A. gracilipes* workers were conducted by teams of people walking in parallel lines, com-mencing in any direction from an *A. gracilipes* detection point. In the absence of definitive knowledge of *A. gracilipes* dispersal distances and population dynamics, the boundary of a supercolony was defined as the last detection point in every direction after which no *A. gracilipes* had been detected for at least 100 m. The figure of 100 m was used due to the direct on-ground finding in both this study and additional unpublished work that 100 m was the greatest distance between detections within supercolonies where colony initiation by budding appears to be undoubtedly the sole dispersal method. This boundary definition did not hold true for some supercolonies larger than 30 ha, but in such cases the more widely-spaced detections were considered to be part of the same supercolony because there was almost no likelihood of human-mediated dispersal (i.e., they were positioned well within an intact environment with no signs of anthropogenic disturbance, as is the case for most of the region), and there were biologically meaningful explanations for these patterns (see later).

Assessments were conducted haphazardly but regularly (approximately one per every 2 m), and each search location was recorded in a GPS unit in categories of either *A. gracilipes* present or absent. The accuracy of visual assessments in determining *A. gracilipes* presence/absence was verified in previous pilot studies, where visual assessments accurately detected the full extent of multiple small and isolated clusters of nests previously delineated using attractive lures (data not presented). Importantly, this visual method did not attempt to detect nests, as it would be an impossible task to complete accurately at the scale of multiple hectares. Rather, it was assumed that workers will be foraging within close proximity to nests, and the foraging distance would be predominantly within the GPS accuracy error margin of +/-10 m.

Cluster analysis was applied to the *A. gracilipes* detections within each supercolony to assess whether supercolonies were comprised of a single contiguous population of ants or multiple smaller-scaled clusters. All spatial analyses were conducted using R ( R Development Core Team 2010 ). First, data were defined as the class “planar point pattern.” The Euclidean distance between each pair of detections was then calculated, and a hierarchical clustering tree plotted. Supercolony 7 was used to determine the clustering cut-off vector value (301) because it was the smallest supercolony that had a detection point almost exactly 100 m from all other detections within the supercolony (the definition of the boundary of a supercolony). All detections within each supercolony were then classified into clusters according to the Euclidean distance threshold of 301.

Two supercolonies (13 and 14; Table 1 ) had very infrequent and widely-dispersed (>100 m) detections. To aid confidence in the accuracy of the non-detections as determined by visual assessments, the absence of *A. gracilipes* between two pairs of greatly-dispersed visual detections within supercolony 13 was assessed using two grids of pitfall traps (plastic containers of diameter 42 mm partly filled with propylene glycol as a preservative) placed in a 5 x 3 array with 10 m spacing between traps. The traps were located approximately midway between the pairs of detections and covered 29% and 17% of the distance between the detections respectively. The traps were operated for 48 hours in October/November each year from 2005 to 2009. The contents of the traps were assessed for the presence of *A. gracilipes* .

**Table 1.**
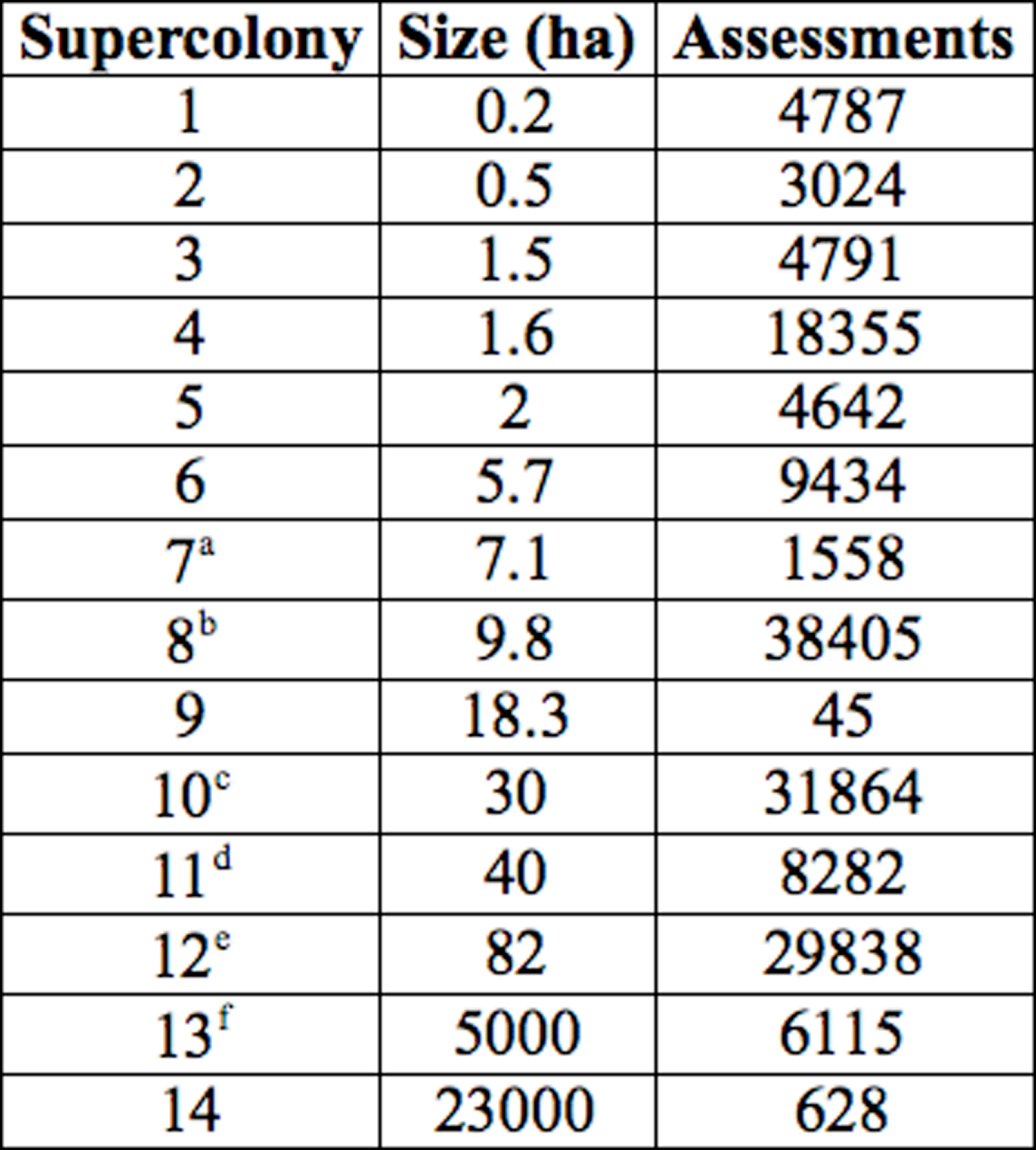
Size and number of assessments made to determine the structure of fourteen supercolonies. Assessments were visual determinations of *Anoplolepis gracilipes* presence/absence. Letters in superscript indicate supercolonies displayed in Figure 2 .

Supercolonies 13 and 14 noticeably supported fewer *A. gracilipes* , so an additional comparison quantifying *A. gracilipes* abundance was made between these two supercolonies and 11 others supporting greater abundances between 8 May and 6 June 2009. Only supercolonies 9–11 were able to be sampled, as the others had undergone toxic treatments, so an additional eight supercolonies undergoing genetic analysis ( Gruber et al. 2012 ) were sampled instead. The abundance of *A. gracilipes* was quantified using the card-count technique utilized on Christmas Island to determine the distribution of high-density populations ( Green et al. 2004 ). Within each supercolony, ant abundance counts were conducted at 11 stations spaced 10 m apart on 100 m transects. Three replicate transects were distributed haphazardly throughout each supercolony, varying from 10 m to approximately 10 km apart. At each station, a laminated card, 10 x 10 cm, divided into four equal quadrants was placed on the ground. For the first 20 seconds, the card was observed to see which quadrant was first contacted by a foraging *A. gracilipes* worker, and this was the quadrant used to count *A. gracilipes* abundance. During the subsequent 30 seconds, the number of ants walking across the selected quadrant was counted. If no ants came in contact with the card during the first 20 seconds, the quadrant first contacted in the subsequent 30 seconds was used and the ant was counted. During counting and as far as practically possible, an individual walking across the quadrant more than once was not counted multiple times. The abundance figure for each supercolony was calculated by pooling the counts of the 11 stations along each transect and then averaging the summed count of the three replicate transects.

### Colony-level intraspecific aggression

Prior lab-based investigations of worker-level interactions have already demonstrated that *A. gracilipes* throughout this region display very little, if any, aggression among supercolonies ( Gruber et al. 2012 ). However, worker aggression is known to be influenced by worker number in other invasive ants ( Holway and Case 2001 ; Roulston et al. 2003 ; Kabashima et al. 2007 ), thus there can be differential outcomes as the scale of observation increases to the colony-level. This experiment investigated colony-level interactions, and tested whether colonies sourced and constructed from different supercolonies would interact non-aggressively and merge together. Artificial nests were constructed of clear plastic containers (23 x 17 x 12 cm) containing a 1 cm ^3^ piece of wet paper. Each of a pair of artificial nests was inoculated with a queen and approximately 50 workers collected from two different supercolonies separated by at least 10 km. The use of queens and workers from different source supercolonies aimed to create conditions for a greater likelihood of individual discrimination and thus aggression, which would decrease the likelihood of nest merging.

All ants had their abdomens marked with a spot of either white or red paint using an Artline 400XF Paint Marker ( www.artlineworld.com ) to distinguish to which colony they belonged. After 24 hours, paired nests were connected using clear Perspex tubing (diameter 55 mm), 250 cm long, approximately corresponding to the mean nest distance found during the nest distribution observations. Following connection, the ants were observed for 30 minutes to infor-mally qualify worker interactions between nests, specifically noting any signs of aggression and to see if the two colonies would merge into a single colony in a single nest box. If the colonies hadn’t merged within 30 minutes, they were re-assessed after 24 hours. This experiment was replicated seven times and conducted from August to October 2007.

### Nest fidelity

Nest fidelity was assessed by quantifying worker abundance changes within artificial nests placed within a field supercolony. A 75 mL plastic container of 42 mm diameter represented a nest chamber and contained a 0.5 cm ^3^ piece of damp tissue paper for moisture. This container had a 2 mm diameter hole in the bottom of the side wall that was big enough to allow workers to pass through, but not queens. The nest chamber was placed into a larger plastic container (all sides 8 cm), which had a 1 cm diameter hole cut into the base of a side to represent a typical *A. gracilipes* nest entrance (B. Hoffmann, personal observation). In the field, the experimental nests were placed within wire cages to prevent disturbance by animals, and the cages were covered with cloth on the eastern side to prevent the artificial nests receiving direct exposure from the morning sun.

The artificial nests were inoculated with queens from a supercolony located 14 km from the trial supercolony and workers from another supercolony located 18 km from the trial supercolony. The two source supercolonies were located 11 km from each other. Just as for the colony-merging trials, the use of queens and workers from different source supercolonies aimed to create conditions with a greater likelihood of individual discrimination and therefore aggression, which would promote fidelity. If *A. gracilipes* displayed high intraspecific aggression and nest fidelity, then both the experimental workers and the workers of the host supercolony would reject the experimental queens, and the workers from the host supercolony would not populate the artificial nests. The opposite would be so for low intraspecific aggression and low nest fidelity. A total of 12 trials were conducted using three treatments: (1) no ants in nest chamber as a control (five replicates); (2) single queen and ten workers to represent an established colony (five replicates); (3) a single queen only (two replicates) to assess whether any dynamics measured in the second treatment were due to the presence of the queen or the workers.

Ants placed in the artificial nest chamber on the first trial day had the top of their abdomens painted white using paint from an Artline 400XF Paint Marker to identify them as the original inoculants. Unmarked ants within the artificial nests in subsequent days were counted and painted red. In the few in-stances that dead ants were found in the artificial nests, they were either not counted if they were not painted, or they were removed from the prior day’s count if they were painted. Ants in both nest containers of each artificial nest were considered together and were all placed in the smaller nest chamber before being returned to the field at the end of each day. Because *A. gracilipes* forage predominantly nocturnally within northeast Arnhem Land due to high daytime temperatures (B. Hoffmann, unpublished data), experimental nests were placed in the field at 17:00 and re-collected the following morning around 09:00 when foraging had ceased. Artificial nests were operated for four days in November 2009 within two ses-sions, with each session testing all treatments simultaneously.

Statistical analyses were not performed, because no ants occupied the control nests (i.e., all data were 0), thereby precluding the ability to conduct a valid statistical test. However, the results of the two treatments versus the control are clear and indisputable.

### Resource flow

Red Envirodye ^®^ (Active constituent Diazo Dyestuff, SST Australia, www.sstaustralia.com ), hereafter referred to as dye, was used to assess resource flow among nests within a supercolony. Preliminary investigations demonstrated that the dye was not toxic to *A. gracilipes* workers and that it could be transferred among workers by trophallaxis. Three variably-sized plots (see below), positioned at least 80 m from each other and with differing *A. gracilipes* densities within a single supercolony, were used sequentially to measure resource flow. Plots were burnt to facilitate locating nests, with plots 1 and 3 burnt one day prior to sampling and plot 2 being burnt 39 days prior. The differing assessment times post-fire was unavoidable, but is not believed to have affected *A. gracilipes* behavior for these experiments. Nests were located by following workers trailing to and from tuna lures (teaspoon of tuna) placed in a grid with 2 m spacing throughout the entire plot. Following mapping, a single nest near the plot periphery was selected as the nest to be supplied with dye, hereafter referred to as the source. The distances from the source to all other nests in the plot were then measured. The source was provided with 15 mL of dye at 17:00 each day for the duration of each experiment, but ants feeding on the dye were not necessarily only from the source nest. Twenty ants were collected from all nests within the plots every morning for the duration of each experiment. The ants were killed in a freezer within half an hour of collection and then dissected and inspected for the presence/absence of dye.

The first plot, which contained the greatest nest density and worker abundance, commenced with dimensions of 20 x 20 m. Sampling did not begin until day nine, by which time the dye had already spread to nests at the periphery of the plot. The plot was consequently expanded to 25 x 25 m on day 11, and then to 40 x 45 m on day 17, when sampling ceased.

The second plot contained intermediate nest density and worker abundance, was 10 x 40 m, and sampling was conducted only for four days. Only nests within 20 m of the source were sampled on the first day, but all nests were sampled on subsequent days. The third plot contained the lowest nest density and worker abundance, was 30 x 35 m, and was sampled for six days.

Following sampling in all three plots, worker foraging density was measured simultaneously in each plot by placing nine pitfall traps (plastic containers with an internal diameter of 65 mm, one third filled with pro-propylene glycol as a preservative) in a 3 x 3 m array with 5 m spacing between traps, located centrally within each plot. The traps were operated for 48 hours, after which time the number of *A. gracilipes* workers in each trap were counted and averaged for each plot. All work was conducted in September and October 2009, when *A. gracilipes* worker and nest densities are at their lowest (B. Hoffmann, unpublished data).

Data were analyzed using binomial General Linear Models using R ( R Development Core Team 2010 ). The number of ants containing dye was expressed as a percentage and used as the dependent variable, distance from the source was the independent variable, and sample time was used as a categorical variable. All models were designed with the categorical variable having a common intercept because in all cases, all ants sampled from the source nest contained dye. The statistical significance of the differences of slopes of the response variable within and between models was tested using an F test.

## Results

### Supercolony structure

**Figure 2.**
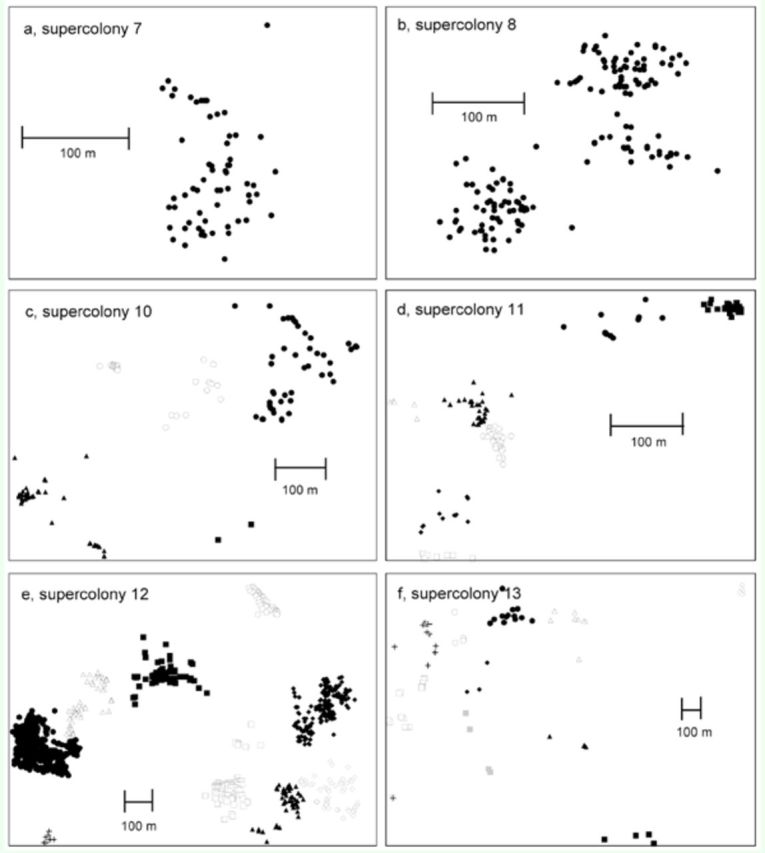
Supercolony structure as determined by *Anoplolepis gracilipes* patterns of occurrence at six of fourteen supercolonies, representing the variation in structure. Symbols are *A. gracilipes* detections from visual searches of the landscape. Different symbols identify statistically distinct clusters as determined by spatial analysis. Note: Figure f displays only a portion of the supercolony. High quality figures are available online.

Supercolonies displayed a continuum of structures from aggregated and contiguous detections in a single cluster, with peripheral detections being more widely separated (up to 100 m apart) ( Figures 2a , b ; supercolonies 1–8 in Table 1 ), to highly diffuse having little continuity ( Figure 2f ; supercolonies 13 and 14 in Table 1 ), with detections often hundreds of meters apart. Detections within all supercolonies greater than 10 ha were separated into multiple clusters within cluster analysis ( Figures 2c–f ).

The absence of *A. gracilipes* between dis-parate detections at supercolony 13 was confirmed by pitfall traps in both assessment plots throughout four years of sampling. Importantly, the two diffuse supercolonies (13 and 14) were so large that mapping their full extent was not feasible, but regional surveys (not detailed here) indicated that supercolony 14 was at least 23,000 ha and supercolony 13 at least 5,000 ha ( Table 1 ). The mean abundance of *A. gracilipes* on card counts within the two diffuse supercolonies was 0.95 ± 1.03 SE, compared with 20 ± 4.20 SE in the aggregated supercolonies.

### Colony-level intraspecific aggression

Aggression was rarely observed between workers from paired nests, and in all cases, paired colonies merged into a single nest box. In one trial, merging occurred within 12 minutes, and all others merged within 24 hours, confirming that the lack of worker-level aggression quantified in prior work corresponded with a lack of colony-level aggression.

### Nest fidelity

**Figure 3.**
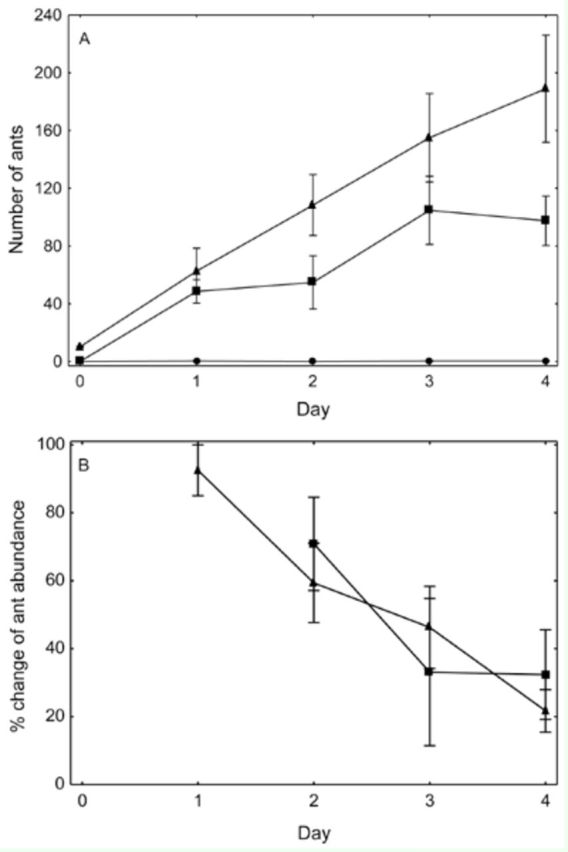
Changes of *Anoplolepis gracilipes* worker abundance (a), and percentage change of workers present in the prior day (b), over four days within artificial nests for three treatments being: nests originally containing one queen and 10 workers (triangle); one queen only (square); or no ants (circle). High quality figures are available online.

Workers displayed low nest fidelity by readily inhabiting the artificial nests, but such a change in nest location was dependent upon the presence of a queen ( Figure 3a ). The two treatments containing queens displayed similar population increases over time, although the treatment that commenced with only a queen had a lower mean population by the final sample day. The original workers in the worker + queen treatment were not present after day 1 in four of the five replicates, and gone by day 2 in the fifth replicate. Low nest fidelity was also demonstrated by the constant turnover of ants within the artificial nests ( Figure 3b ). Initially, when overall populations in each nest were low, the turnover was over 90%, but this turnover rate decreased linearly, irrespective of treatment, as the populations increased.

### Resource flow

**Figure 4.**
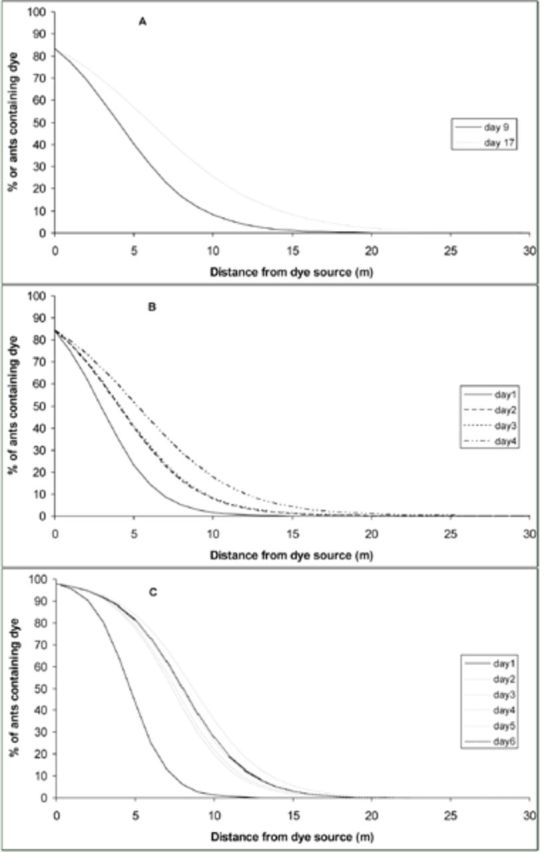
Binomial General Linear Models of the relationship between the percentage of *Anoplolepis gracilipes* workers sampled containing dye with the distance of each nest sampled from the dye source at three plots (figures a–c respectively). High quality figures are available online.

The three plots displayed a range of nest densities and mean number of workers per pitfall trap, with greatest values in plot 1 and lowest in plot 3 ( Table 2 ). In all plots and for all sample times, there was a negative relationship between the distance from the source and the uptake of dye ( Figure 4 ). The dye was detected up to 13 m within 24 hours, and was found as far as 32 m after four days ( Table 2 ). The rate of spread of the dye through each plot, as well as the distance the dye travelled, was clearly affected by the ant density of each plot, with the plots with greater ant density (plots 1 and 2) having dye dispersed farther and quicker than the low density plot ( Table 2 ). Both the distance that dye was dispersed away from the source and the percentage of workers in each nest containing dye were positively related to time, resulting in successive models with time having lower slopes and longer tails as well as being significantly different (Plot 1, F = 166, *P* < 0.001; Plot 2, F = 92, *P* < 0.001; Plot 3 F = 74, *P* < 0.001; Figure 4 ). In only the low-density plot did the models stabilize, indicating that the dye was not being dispersed farther through the plot, and that the percentage of workers in each nest containing dye was not changing ( Figure 4 ).

**Table 2.**
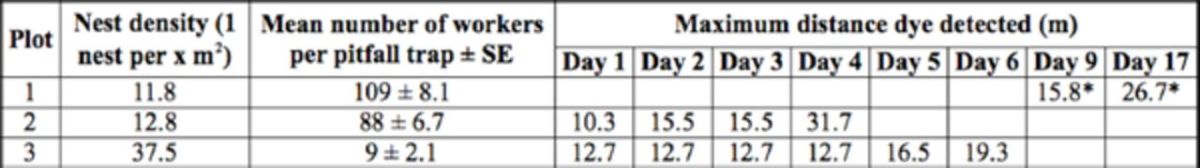
*Anoplolepis gracilipes* measurements and maximum distance dye was detected on each sample day for each plot. * indicates that samples were unlikely to have covered the full extent of the distribution of the dye.

## Discussion

### Supercolony structure

Despite *A. gracilipes* being considered a species that solely uses budding as its dispersal strategy, supercolonies larger than 10 ha were not comprised of a single contiguous unit of ants, but rather multiple clusters. Also, there was a clear reduction in cluster size and density between the numerous relatively small supercolonies (1–12) and the two large supercolonies (13 and 14). Biotic resistance has already been discounted as a driver of *A. gracilipes* distribution and abundance patterns within Arnhem Land ( Hoffmann and Saul 2010 ), and such patterns within the large supercolonies are too large to correspond to potential seasonal variation of expansion and contraction (e.g., Heller et al. 2006 ). Ant species and communities can indeed be sensitive to minor habitat differences ( Tschinkel et al. 2012 ), however, the range of abiotic variation of this environment is well within the known limitations of this ant, and the limited abiotic heterogeneity presented by this environment does not correspond with the dramatic broad-scale variations displayed both within and among supercolonies. Almost 100 supercolonies of this ant were mapped in this region, and abiotic factors did not appear to be a principal force behind these patterns. Habitat and resource heterogeneity no-doubt play a role at a fine scale (tens of meters), but I propose that the interplay of up to three factors drives patterns at the clustering scale (>100 m).

The first factor is supercolony age. It is reasonable to assume that there is a positive relationship between the age of a supercolony and its size. Although the age of each supercolony is not known, the largest (supercolony 14) probably exceeds 70 years ( Young et al. 2001 ). The clear pattern with increasing supercolony size (and thus presumably age) is for an increase in the number of clustered nests, smaller cluster size, greater spatial separation of clusters, and reduced *A. gracilipes* abundance. These trends are consistent with a common temporal sequence of ant invasions being a population “explosion” soon after invasion, later followed by a population decline, fragmentation, and even extinction ( Haines and Haines 1978b ; Morrison 2002 ; Wetterer et al. 2006 ; Cooling et al. 2012 ). Thus it is likely, at least for the two largest supercolonies (13 and 14), that much of their internal fragmentation represents relics of a formerly continuous distribution of nests, resulting from population decline to a level of ecological equilibrium.

The second factor is dispersal method. Although it is clear that *A. gracilipes* initiates new nests predominantly by budding ( Haines and Haines 1978a ; Rao et al. 1991 ; Haines et al. 1994 ), nuptial flights are known to occur ( Haines and Haines 1978a ; Abbott 2006 , personal observation). It is unclear, however, if newly-inseminated queens are able to found a new colony independently or if they must join an existing colony. The presence of isolated nests located 100 m or more from all other nests ( figures 2a , c , f ) suggests that this ant can indeed initiate independent colonies following a nuptial flight. It is quite possible, therefore, that some extent of clustering is a result of dispersal events via flight, with subsequent dispersal by budding. Other ants utilize both dispersal strategies ( Rosengren et al. 1993 ; Tschinkel 2006 ), with the relative contribution of both reproductive strategies changing relative to habitat heterogeneity and suitability ( Molet et al. 2008 ). This reproductive flexibility is believed to allow a species to be more competitive in a more diverse array of habitats than they would be using a single reproductive mode.

The third factor likely to determine patterns of clustering is genetics. When species are accidentally dispersed outside of their native range they experience a genetic bottleneck ( Taylor and Hastings 2005 ). Such genetic restriction clearly does not necessarily become a constraint on a species, as many ants have become highly successful invaders ( Holway et al. 2002 ). The reasoning for their success is attributed to a shift to, or an increase in, polygyny and supercoloniality, corresponding with a change in dispersal tactic from nuptial flights to budding ( Helanterä et al. 2009 ). Theoretically, subsequent long-distance dispersal events will further increase the genetic bottleneck and strengthen the resultant syndromes. It is therefore possible that the largest diffuse supercolony with low ant density (supercolony 14) is the initial incursion point and the many smaller and more aggregated infestations with high population density arose from later human-assisted long-distance dispersal events, which further increased the genetic restriction (see Gruber et al. 2012 ).

### Nest fidelity

Like *L. humile* , *A. gracilipes* displayed low nest fidelity, and hence flexible nesting behavior, with all artificial nests containing queens from distant supercolonies being rapidly colonized by unrelated workers. Notably, immigration of ants into the artificial nests was not nest migration whereby the entire populous of a nest changes nest location, because queens from the host supercolony were never found in the outer nest chamber, and brood (pupae) were rarely found.

The fate of the original workers in the worker + queen treatment is unknown, but given the lack of aggression displayed in multiple trials presented here, and that it took two days for all of these workers to be lost from the experiment, it is most likely that they merely migrated to nests within the host supercolony, just like many workers from the host supercolony were migrating to the artificial nests. While this experiment does not demonstrate that all workers within a nest will translocate, it clearly showed that many will.

So the question remains, why weren’t these workers that migrated faithful to one nest or to their parental queen? Ants are known for their navigational abilities, so it is unlikely that the workers were merely lost. Whatever the reason, the success of this strategy suggests that perceived negative repercussions of such behaviour, such as reduced relatedness of nest mates, are outweighed by the positive implications that make supercoloniality so successful. Investigations into the consequences of low nest fidelity for an individual, for a population, and for a species remain to be conducted, but will likely be a fruitful future research focus.

The daily turnover rate of workers within a nest cannot be determined here, as turnover had not stabilized after four nights. However, given that nests in Arnhem Land usually contain >500 workers (B. Hoffmann, unpublished data), being more than double the mean artificial nest population after four days, the daily turnover rate is likely to be well below the 20% figure reached by the end of the experiment. In the only other publication quantifying worker turnover between nests within a supercolony, Markin (1968) found turnover within an *L. humile* nest exceeded 50% in one week.

An additional chance observation of this experiment was the frequent presence of callow workers within the artificial nests. Although this may simply be a result of recruitment, the lack of brood within the artificial nests, and hence the lack of need for brood-caring, may also indicate that *A. gracilipes* does not display age-dependent task allocation, whereby young workers perform the safe internal nest tasks, and older workers forage ( Mirenda and Vinson 1981 ).

### Resource flow

Due to the low nest fidelity displayed by *A. gracilipes* , coupled with trophallaxis, it was not surprising that the dye was dispersed to nests far from the source. Resources would not be expected to flow through the entirety of a supercolony, as in most cases the size of the supercolony is far too great to expect any interaction between individuals at opposite ends. Indeed, here resources were found maximally 32 m from the source, yet supercolonies with continuous detections could be approximately 300 m wide (10 ha). Clearly, however, resources are capable of being dispersed great distances relative to the size of an ant throughout a supercolony. The obvious benefit to supercolonial ants is that spatially restricted resources are made available to, and benefit, a much wider population than just the individuals from nests closest to a resource. This is surely a mechanism promoting invasion success of supercolonial ants, in addition to typically superior abilities to usurp resources ( Human and Gordon 1996 ; Holway 1999 ).

Dye was detected up to 32 m away from the source within four days, and it is very likely that resources can be dispersed much farther, especially over longer periods. Most other related research on other supercolonial invasive ant species have also found resources rapidly move to the edge of sampling areas, thereby preventing quantification of the maximum distance that resources move with time. For *L. humile* , Markin (1968) detected resources 49 m away in 24 hours, Ripa et al. (1999) detected resources at 33 m after 24 hours and 54 m after 72 hours, and Vega and Rust (2003) found that over 50% of ants contained marker dye at 61 m after two weeks. Likewise for *P. megacephala* , Mortreuil and Brader (1962) detected a resource in individuals at 8 m in 24 hours.

The only exception to date of resource tracking within strongly supercolonial species not being limited by plot size is the study of *L. humile* by Heller et al. (2008) in northern California. In this study, labeled ants were not found beyond 50 m due to the novel finding of spatially limited interacting nests. In my study, no evidence of such colony-groups was found, although it may occur at a greater scale.

Of the other species studied, none have displayed resource sharing among nests within a supercolony to the extent as has been shown for *L. humile* and now *A. gracilipes* . Buczkowski and Bennett (2006) found that that a resource was only detected in workers of *T. sessile* utilizing the trail being provi-sioned with the resource, but not at unconnected locations often closer to the feeding location. Likewise, nests of polygyne *S. invicta* only share limited resources with neighboring nests ( Drees et al. 1992 ; Weeks et al. 2004b ) and do not share queens among nests that do not have a former connection, at least in summer ( Vargo and Porter 1989 ).

Finally, it appears likely that resource flow is dependent upon ant and nest density. Resources moved farther and quicker in the two plots with the greatest nest and forager ant densities, most likely due to the greater population levels being able to provide greater turnover of individuals among nests, as well as the closer proximity of nests allowing easier exchange of workers among nests. Resources in plot 3, the plot that contained the lowest nest density and forager abundance, were not detected in the farthest nests, where nest separation exceeded seven meters. This is consistent with patterns of food-flow among neighboring polygyne *S. invicta* nests, which are also known to be partly determined by the distance among colonies ( Weeks et al. 2004b ).

### Conclusions

This study has clearly demonstrated that *A. gracilipes* strongly displays these supercoloniality traits, and for the first time has detailed the structure of entire supercolonies for any invasive supercolonial ant. The structure of *A. gracilipes* supercolonies varied markedly, with those smaller than 10 ha being comprised of a single contiguous population, and larger populations displaying increasing fragmentation. Likewise, the smaller supercolonies comprised greater ant densities than the largest ones. Workers were not faithful to nests or queens, with a daily turnover rate remaining unclear but being below 20%. Queens were readily accepted from distant supercolonies, and resources were readily and rapidly moved among nests, with the rate and distance of resource movement being relative to worker and nest density.

Quantification of the expression of traits underlying supercoloniality is required for other species to improve our understanding of this social strategy. This is especially so for invasive ants, as there are only a handful of highly invasive and ecologically dominant ant species, and supercoloniality is a trait common to them all. Until now, only the supercolonial traits of *L. humile* and *S. invicta* have been studied in detail, and partially at that. This research has demonstrated that *A. gracilipes* strongly displays all supercolonial traits, comparable to *L. humile* . Further research will likely aid our understanding of factors promoting invasion success, potentially with implications to improve management.
